# The Effects of Intensive Nutrition Education on Late Middle-Aged Adults with Type 2 Diabetes

**DOI:** 10.3390/ijerph13090897

**Published:** 2016-09-08

**Authors:** Ye Li, Meihong Xu, Rui Fan, Xiaotao Ma, Jiaojiao Gu, Xiaxia Cai, Rui Liu, Qihe Chen, Jinwei Ren, Ruixue Mao, Lei Bao, Zhaofeng Zhang, Junbo Wang, Yong Li

**Affiliations:** 1 Department of Nutrition and Food Hygiene, School of Public Health, Peking University, Beijing 100191, China; lydia30130@163.com (Y.L.); xumeihong@bjmu.edu.cn (M.X.); rfcaubj@126.com (R.F.); jiaojiaogu442@gmail.com (J.G.); lrui0101@163.com (R.L.); qiheyuntian@163.com (Q.C.); ren_jinwei@126.com (J.R.); rx334@163.com (R.M.); zzfeng1104@bjmu.edu.cn (Z.Z.); bmuwjbxy@bjmu.edu.cn (J.W.); 2 Department of Nutrition, China-Japan Friendship Hospital, Beijing 100029, China; maxt2008@sina.com; 3 Department of Nutrition and Food Hygiene, School of Public Health, Capital Medical University, Beijing 100069, China; shuiruoran8886@126.com; 4 Department of Clinical Nutrition, Peking University International Hospital, Beijing 102206, China; baolei6230@163.com

**Keywords:** education, nutrition, diabetes mellitus, type 2, late middle-aged adults

## Abstract

*Objective*: Many patients with type 2 diabetes find it difficult to maintain good glycemic control. Undesirable glycemic control occurs greatly due to deficiencies of nutritional knowledge and difficulty in obtaining dietary prescriptions. The late middle-aged and elder individuals are the main populations that are affected by type 2 diabetes. The main purpose of this study was to investigate whether intensive nutrition education would make benefits for late middle-aged patients with type 2 diabetes. *Method*: 196 patients between 50 to 65 years old meeting type 2 diabetes criteria and eligible for the program were included in a single-blinded, 30-day centralized management of an education program in China. Participants in the program were randomly divided into a usual nutrition education group or an intensive nutrition education group. The usual nutrition education group was used as a control group and received only basic health advice and principles of diabetic diets at the beginning and the end of the study. Participants in the intensive nutrition education group were arranged to receive intensive nutritional lectures about diabetes for 30 days. The primary outcomes were the changes in weight, body mass index (BMI), fasting plasma glucose (FPG), 2-h postprandial plasma glucose (PG), glycosylated hemoglobin (HbA1c), total glycerin (TG), total cholesterol (TC), high-density lipoprotein cholesterol (HDL-c), and low-density lipoprotein cholesterol (LDL-c). *Results*: After 30 days of intervention, FPG, PG, and HbA1c in the treatment group decreased significantly than the control group (*p* < 0.05). HbA1c reduced significantly by 0.6% in the intervention group. No significant differences in the change of blood lipids were observed between groups. However, TG, TC, and HDL-c made improvements compared with the baseline in the experimental group. Both groups had a reduction in weight and BMI within groups, especially in intensive nutrition education group. However, there was no statistical significance between groups. *Conclusions*: Intensive nutrition education has significant effects on blood glucose control in late middle-aged adults with type 2 diabetes. Intensive education can cultivate good diet habits and increase physical activity, which are important for diabetes patients in the short and long terms. These findings may contribute to improving education methodology and nutrition therapy in patients with type 2 diabetes.

## 1. Introduction

Diabetes has become a major public health problem for the global increasing prevalence of diabetes. There will be 366 million people with diabetes in 2030, a growing public health concern across the world [1]. Therefore, robust strategies aimed at the prevention and treatments of diabetes are needed. Diabetes nutrition therapy is one of the most important strategies for diabetics [2]. Undesirable glycemic control occurs greatly due to deficiencies of nutritional knowledge and difficulty in obtaining dietary prescriptions [3]. Education is an effective method to make up for the deficiency of knowledge. Several trials have shown that education can improve knowledge and practice of patients, which were helpful in achieving positive clinical, lifestyle, and social outcomes [4,5,6,7,8]. However, many patients with type 2 diabetes find it difficult to maintain lifestyle changes [9]. Further work is needed to investigate more effective types of education towards diabetes control.

Late middle-aged and elder individuals are the main population that is affected by type 2 diabetes [9]; late middle age is an especially important period for the prevention of complications in old age. However, few studies have illuminated the ideal characteristics of education programs in late middle-aged patients with diabetes. Especially, education themes and frequency should be checked for optimal results. The aim of this program is to explore the effects of an intensive nutrition education program on older middle-aged adults with type 2 diabetes, especially the impact of improving lifestyle and attaining glycemic, lipid goals.

## 2. Methods

### 2.1. Ethics Statement

This study protocol was approved by the ethics committee of China-Japan Friendship Hospital of Ministry of Health of the People’s Republic of China (Approval Number: No. 2011-93) and complied with the Second Declaration of Helsinki.

### 2.2. Study Design and Participants

This was a randomized controlled trial conducted at a study center in Baotou, Inner Mongolia Autonomous Region, China. Two hundred patients meeting type 2 diabetes criteria (based on the WHO criteria) were recruited via advertisements. Eligible participants were adults aged between 50 to 65 years old, and met the following criteria: fasting plasma glucose (FPG) ≥7.0 mmol/L, 2-h postprandial blood glucose (PG) ≥11.1 mmol/L, and glycated hemoglobin (HbA1c) >7%. Any type of glucose-lowering medication could be used, but the medication should be stable. Exclusion criteria were severe renal or hepatic complications, receiving glucocorticoid treatment, having malignancy during the preceding 3 months or other factors that may exert influences to the trial. Those who had participated in any other diabetes education program recently were excluded.

**Figure 1:**
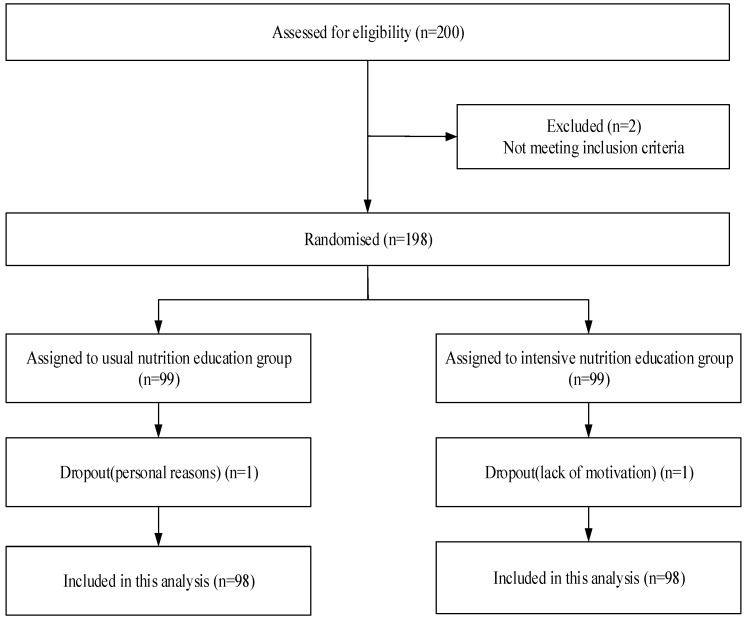
Flow of participants though trial.

Of the 200 recruited diabetes patients, 198 were eligible for the program and enrolled in the study through face-to-face assessment and examination. The reasons for exclusion were serious complications and glycated hemoglobin lower than 7%. Eligible participants were randomized by computer using a random number method: 99 participants were assigned to the usual nutrition education group and 99 to the intensive nutrition education group. Before the commencement of the education program, informed consent was signed on a volunteer basis. There was one dropout in each group during the first week of the 30-day study. Finally, 196 participants were included in the analysis (Figure 1). Adverse events were not reported during the program.

### 2.3. Interventions

Participants were randomly assigned to a usual nutrition education (UNE) group or an intensive nutrition education (INE) group. The usual nutrition education group was a control group and received only basic health advice and principles of diabetic diets at the beginning and the end of the study. Participants had meals following their own diet habits. The intensive nutrition education group were arranged to live together for 1 month to received intensive nutritional lectures about diabetes. Diets were provided for the meal time and participants took food under guidance. Except for the collective class time and meal time, participants in the INE group could schedule their own time as usual during the program.

To promote healthy eating habits appropriate for diabetes, participants in the INE group were given nutrition education classes every morning for 30 days. The education was a multidimensional nutritional guidance. The contents of education included basic knowledge of diabetes, nutrition equilibrium, nutritious diet design, exercise techniques, blood sugar monitoring, and medication treatment of diabetes (Table 1). Education providers were professional health educators, doctors, and nutritionists. After classes, discussion periods would be arranged for the communication of participants and explanations of questions.

Meals were served at the study center restaurant, which specialized in traditional Chinese dishes. We provided a variety of food including cereals, vegetables, legumes, fruits, meats, fish, eggs, and dairy. Diet was supplied in the form of a buffet. Nutrients of each dish were calculated for further analysis of participants’ daily intake. Participants could choose food to their own tastes, while guidance was given on choosing proper diet suitable for diabetics. Participants were encouraged to choose food of lower glycemic index, energy density, and fat content. Types and quantities of the food eaten by participants were recorded by our staff.

The program not only provided theories of diabetes and nutrition, but also advised as to the application of the diabetic and nutritional knowledge learned from this education project. With education and site guidance, participants interpreted and applied the knowledge to build healthier diet habits for diabetes. Furthermore, the education program recommended 30-min medium strength exercises per day by the participants’ own choice. Participants could choose any type of medium physical excises as learned from education classes voluntarily, such as walking, dancing, gymnastics, and tai chi.

### 2.4. Data Collection

To evaluate the effectiveness of our education program, the assessment of diabetes-related knowledge was performed. Scores for knowledge were assessed by questionnaire and face-to-face interviews. The knowledge instrument used was designed on the basis of the English version of Diabetes Knowledge Questionnaire (DKQ) [10]. The Chinese DKQ of our program has been tested for reliability and validity. The DKQ included information about respondent’s understanding of the diagnosis of diabetes, complications of diabetes, balanced diet, and self-management skills. Responses were graded as “yes,” “no,” or “don’t know”. The final score was based on the percentage of correct scores, with a maximal possible score of 100. Eligible patients were taken to a private area in the study center to complete the study questionnaire. Participants completed the assessment before and after the scheduled education. Food intakes and physical activity of the participants were estimated by the diet record and the International Physical Activity Questionnaire (IPAQ). Furthermore, participants were required to record their medication, fingertip blood glucose if tested, and uncomfortable situations.

Weight and height were examined comprehensively. Participants were required to fast for at least 12 h before the blood collection. Venous blood samples were tested for the following measurement: fasting plasma glucose (FPG), 2-h postprandial plasma glucose (PG), total glycerin (TG), total cholesterol (TC), and high-density lipoprotein cholesterol (HDL-c), which were measured by the enzymic method; low-density lipoprotein cholesterol (LDL-c), which was calculated; glycosylated hemoglobin (HbA1c), which was measured by the high-performance liquid chromatography method.

The primary outcomes were the changes in fasting and 2-h blood glucose, HbA1c, and other lipoprotein parameters. Assessments were conducted at baseline and at the end of the 30-day intervention. All medical inspections were undertaken by certified clinic staff (Inner Mongolia Medical College, Third Hospital, Baotou, China) at the same laboratory.

### 2.5. Statistics

The study was powered to detect differences in the primary outcome (HbA1c concentration) between the usual nutrition education group and the intensive nutrition education group 30 days after randomization. We assumed an SD of 1.6% for HbA1c, on the basis of reported values among patients with type 2 diabetes, and sought a target difference of 0.7%. The sample size was based on 80% power with a 5% two-sided α to detect the above differences, allowing for 15% of missing data.

Descriptive statistics were conducted to describe sample characteristics with means (standard deviation, SD) for continuous variables or numbers (percentage) for categorical variables. Equality and normality of variance were checked before any further analysis. Data were checked for normality by using Kolmogorov–Smirnov values. Independent sample *t*-test and the χ^2^-Test were used to examine significant differences between groups. The paired Student’s *t*-test and the Wilcoxon test were used for within-group comparisons. Data were analyzed on an intention-to-treat basis. All analysis was performed with the Statistical Packages for the Social Sciences (SPSS-19.0) software (SPSS Inc., Chicago, IL, USA). A *p-*value of less than 0.05 was considered significant.

## 3. Results

### 3.1. Basic Characteristics of the Study Population

Characteristics of the participants enrolled in this study, as stratified by groups, are shown in Table 2. The features of participants assigned to the two groups were similar at baseline (*p* > 0.05).

### 3.2. Diabetes Knowledge, Behavioral, and Medication Outcomes

Data for diabetes knowledge questionnaire, daily dietary intake, exercise, and medication changes are presented in Table 3. Total score of the Chinese Diabetes Knowledge Questionnaire in the INE group was higher than that in the UNE group. The average daily intake of energy was lower and more dietary fiber was taken in the INE group after the 30-day intervention. At the end of the study, participants in the INE group spent 4.5 h being sedentary and 46.8 min in MVPA, while participants in the UNE group spent 6.1 h being sedentary and 23.6 min in MVPA per day. Medication changes showed no statistical significance.

### 3.3. Fingertip Blood Glucose of INE Group

**Figure 2:**
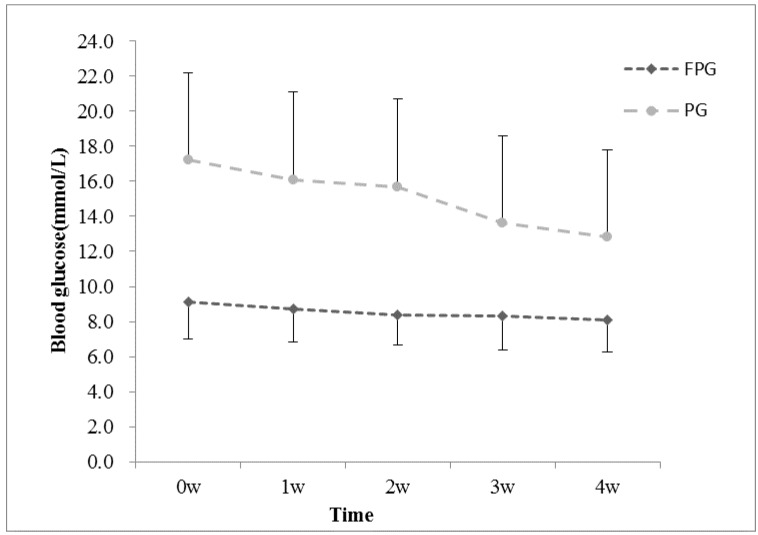
Fingertip blood glucose of INE group. Abbreviations: FPG: fasting plasma glucose; PG: 2-h postprandial plasma glucose.

Blood glucose testing was an important part of diabetes self-care behaviors. Resulting from the self blood glucose record, 84 (85.7%) participants in the INE group monitored blood sugar by themselves more than once a week, while only 28 (28.6%) participants in the UNE group had regular self-monitoring of blood glucose (SMBG). SMBG results of the INE group are shown in Figure 2.

### 3.4. Weight, BMI, Blood Glucose, and Lipid Changes after Intervention

Changes from baseline to 30 days are shown in Table 4. Both groups had reduction in weight and BMI within groups, especially in the INE group. However, there was no statistical significance between groups. After 30 days intervention, the fasting plasma glucose, postprandial blood glucose, and the HbA1c level in the treatment group were lower than the control group (*p* < 0.05). No significant differences in the change of blood lipids were observed between groups. However, TG, TC, and HDL-c made improvements in the experimental group.

## 4. Discussion

In this study, we found that a 30-day, intensive education program had a significantly beneficial effect on glycemic control in late middle-aged patients with type 2 diabetes. Fasting plasma glucose, postprandial blood glucose, and HbA1c level in the treatment group were lower after the intensive education intervention than the control group. Although weight, BMI, and blood lipid did not manifest differences between the two groups, participants in the INE group made improvements in body-weight, BMI, TG, TC, and HDL-c compared with baseline conditions after intervention.

In our study, mean reduction in HbA1c was 0.6% in the intervention group. Former research showed that diabetes self-management education can improve HbA1c and body weight in patients with type 2 diabetes [11]. A recent review found that engagement in diabetes education led to a statistically significant decrease in HbA1c levels, especially a combination of group and individual education results in the largest decreases in HbA1c [12]. Glycemic control is important for the prevention of complications in individuals with T2DM [13]. In our study, an improvement in the blood glucose was observed, but no significant difference was found between groups in other variables, including blood lipids and weight. This may be concerned with the short period of intervention. However, the majority of life style weight-loss programs in adults with type 2 diabetes led to weight loss <5% and did not result in advantageous metabolic outcomes [14].

More diabetes knowledge through behavior was related to glycemic control [15]. To interpret the effectiveness of the intervention, more knowledge was gained from intensive education, and the pattern of nutrition education might have some superiorities. Methods to deliver patient education could be categorized into at least three types including education during usual care, structured group education, and individual (one-to-one) education [4]. Our education program made the attempt to combine group education with individual education. The educators not only gave classes collectively, but also provided individualized guidance. During the 30 days, participants received concentrated and systematic lectures about diabetes in order to remember well and apply the knowledge to real life. The lectures were more frequent and intensive than previous reports. Studies showed that patient participation were of importance for health services. Adequate time should be provided for patients and health professionals to build relationships and share knowledge [16]. In this intensive education program, more chances and time were provided for health educators and patients to interact with each other. Not only were sufficient knowledge about diabetes control presented through lectures every day, but discussion opportunities were also provided after class for communication with educators and among patients. Participants had opportunities to establish excellent mutual-trust relationships with educators through intensive education. The congregate active atmosphere encouraged everyone to take their disease under control. The group motivation might contribute to the confidence and initiative of patients against diabetes.

The main theme of this education program was nutrition guidance and application. In some studies, meal preparation training or diabetes cooking courses led to improvements in eating habits, glycemia reductions, and blood pressure lowering effects [17,18,19]. Favorable dietary changes would be conductive to health benefits [20]. In the present study, diet principles and reasonable food choice were explained for the better collocation of diets at each meal. Low-energy, low-fat, and high-fiber diets were recommended. A nutritionist gave guidance on the right portions of each food group to choose during mealtimes. Total daily average energy intake of the INE group was 2112 kcal, of which carbohydrate provided 60% energy intake, fat 22%, protein 18%, and 31 g of dietary fiber. Participants in the INE group made healthier food choices and consumed more dietary fiber than the UNE group. The centralized management was convenient for the patients to consult momentarily and change poor dietary habits gradually. After the 30-day cultivation, healthy eating habits that would make long-term benefits were developed through nutrition education and dietary recipe practice.

Our diabetes education program targeted not only the growth of knowledge, but also effect behavior change. Nutrition and physical activity are core factors for diabetes. Glycemic control can be improved through nutrition and exercise interventions [21]. Exercise education and guidance were involved in the intervention, and 30-min medium strength exercise per day was recommended. From our observations, participants in the INE group spent more time doing exercise than the control group. A total of 59.2% participants of the intensive education group achieved the exercise goal. MVPA time increased while sedentary time decreased after intervention. Self-monitoring of blood glucose also performed better in the INE group. Furthermore, these behaviors may be influential to glycemic control.

A review suggested that diabetes self-management behaviors were affected by a wide range of personal and environmental factors [22]. Health care professionals should play crucial roles in improving the education and training in nutrition and physical activity because they are unique to stimulate patients’ motivation and achieve good outcomes [23]. Probably benefited by the collective environment, participants in the intervention group were more enthusiastic than those in the control group to take physical exercises and make behavioral changes. Such intensive education is likely to be more effective in producing behavior change than scanty education programs alone. There might be some difficulties for collective living and receiving intensive education under different conditions in other countries. However, if people were willing to participate and were easily gathered together, intensive nutrition education would be applicable and beneficial. In China, more elderly people have chosen to live collectively in senior apartments or rehabilitation centers, and intensive education would be suitable for them.

This intensive type of nutrition education offered a new model of health education. Different from other general life-style intervention, we focused on nutrition knowledge practice and healthy diet habits cultivation, which were the great barriers of diabetes control. Furthermore, the research subjects were late middle-aged adults because late middle age was an important period for preventing diabetes complications and usually affected by memory decline. Few previous education intervention studies have focused on this population. Continuously reinforcing education day-to-day could help imprint memories and achieve good results. Beside the strengths, the trial had some limitations. Owing to the regional restriction in the program, the results presented here are relevant for Chinese people only. Future trails might help to establish the generalizability of the program for patients with type 2 diabetes from varying cultural backgrounds.

## 5. Conclusions

We found that intensive nutrition education had excellent effects on blood glucose control in late middle-aged adults with type 2 diabetes. Intensive nutrition education as the core contents of health education was effective on diabetes control in a 30-day period, working to cultivate good habits and improve self-care abilities. These findings may contribute to the improvement of education methodology and nutrition therapy in patients with type 2 diabetes. More research should be done to evaluate the effects outside of China and the long-term effects of intensive nutrition education.

## Figures and Tables

**Table 1:** Contents of the diabetes nutrition education.

	Contents
Knowledge of diabetes	Diagnosis of type 2 diabetes mellitus
	Symptoms of type 2 diabetes mellitus
	Main causes of type 2 diabetes mellitus
	Complications of type 2 diabetes mellitus
Diabetes medication	The importance of taking medications
	Different types of diabetic medications
	The proper methods of taking diabetic medication
Blood glucose monitoring	The importance of regular blood glucose monitoring
	The optimal target range for blood glucose control
	Methods of self-monitoring of blood glucose
	Techniques of handling abnormal blood glucose
Healthy diet	Balanced diet for type 2 diabetics
	Appropriate caloric intake for type 2 diabetics
	Diet combination and cooking method for type 2 diabetics
	The importance of eating vegetables, fruits and whole grains
Healthy lifestyle	The importance of regular exercise for type 2 diabetics
	The appropriate exercise for type 2 diabetics
	Taking suitable exercises according to personal fitness level
	Giving up bad habits and maintaining healthy behaviors

**Table 2:** Baseline characteristics of the study participants according to group.

Characteristic	Intervention Assignment		*p-*Value
	UNE (*n* = 98)	INE (*n* = 98)	
Male	52 (53.1)	47 (48.0)	0.48
Age (years)	58.3 (4.1)	59.1 (4.6)	0.23
Duration of diabetes (years)	6.5 (5.4)	6.8 (4.8)	0.63
Diabetes medication use			
No diabetes medication	16 (16.3)	14 (14.3)	0.96
Oral medication only	48 (49.0)	49 (50.0)	
Insulin only	17 (17.3)	16 (16.3)	
Oral medication and insulin	17 (17.3)	19 (19.4)	
Education			
Illiteracy	7 (7.1)	2 (2.0)	0.24
Primary school	9 (9.2)	9 (9.2)	
Middle school	25 (25.5)	31 (31.6)	
High school	29 (29.6)	36 (36.7)	
College	28 (28.6)	20 (20.4)	
Height (cm)	164.5 (7.1)	163.9 (8.1)	0.56
Weight (kg)	68.3 (10.0)	69.1 (11.5)	0.64
BMI (kg/m^2^)	25.1 (1.8)	25.7 (3.7)	0.26

Abbreviation: BMI: body mass index. Variables are reported as mean (standard deviation) or number (percent).

**Table 3:** Diabetes knowledge, behavioral, and medication outcomes.

Variable	UNE (*n* = 98)		INE (*n* = 98)		*p-*Value *
	Baseline	30-Day	Baseline	30-Day	
Diabetes Knowledge Questionnaire, mean(SD)					
Total score	53.6 (18.2)	61.8 (19.5)	56.4 (16.4)	84.5 (12.8)	<0.001
Daily dietary intake					
Energy intake (kcal)	2486 (307)	2458 (396)	2452 (253)	2112 (274)	<0.001
Protein(% of energy intake)	19%	19%	17%	18%	0.043
Carbohydrate (% of energy intake)	43%	44%	46%	60%	
Fat (% of energy intake)	38%	37%	37%	22%	
Fiber (g)	19 (6)	22 (5)	21 (4)	31 (6)	<0.001
Exercise					
MVPA (min per day)	23.3 (20.4)	23.6 (18.2)	26.7 (21.3)	46.8 (14.6)	<0.001
Sedentary time (hours per day)	5.6 (2.4)	6.1 (4.4)	5.8 (3.2)	4.5 (3.8)	0.007
30-min MVPA per day, no. (% patients)	31 (31.6)	37 (37.8)	28 (28.6)	58 (59.2)	0.003
Glucose-lowering treatment, no. (% patients)					
Decrease		2 (2.1)		5 (5.1)	0.199
Increase		9 (9.1)		4 (4.1)	
No change		87 (88.8)		89 (90.8)	

Abbreviation: MVPA: moderate to vigorous physical activity; * *p*-values are for the difference between the groups at the end of the study.

**Table 4:** Means of baseline, 30-day changes in variables of participants according to group.

Measure	UNE (*n* = 98)				INE (*n* = 98)				*p-*Value ^‡^
	Baseline	30-Day	Change	*p*-Value ^†^	Baseline	30-Day	Change	*p-*Value ^†^	
Weight (kg)	68.3 (10.0)	68.2 (10.2)	−0.1 (1.0)	0.226	69.1 (11.5)	68.3 (10.6)	−0.8 (2.9)	0.012	0.625
BMI (kg/m^2^)	25.1 (1.8)	25.0 (1.9)	−0.1 (0.4)	0.169	25.7 (3.7)	25.3 (3.4)	−0.3 (1.1)	0.020	0.631
FPG (mmol/L)	9.3 (0.5)	9.2 (0.5)	−0.0 (0.2)	0.673	9.4 (3.1)	8.3 (2.8)	−1.1 (2.4)	<0.001	<0.001
PG (mmol/L)	18.5 (2.4)	18.1 (2.3)	−0.4 (0.9)	<0.001	17.9 (5.6)	15.6 (4.7)	−2.3 (3.9)	<0.001	<0.001
HbA1c (%)	7.9 (0.4)	8.0 (0.5)	0.1 (0.4)	0.224	7.9 (0.4)	7.3 (0.9)	−0.6 (0.9)	<0.001	<0.001
TG (mmol/L)	1.6 (0.5)	1.6 (0.6)	0.02 (0.2)	0.305	1.8 (0.9)	1.6 (0.9)	−0.2 (0.9)	0.008	0.252
TC (mmol/L)	5.3 (1.1)	5.2 (1.1)	−0.1 (0.2)	<0.001	5.1 (0.9)	5.0 (1.0)	−0.1 (0.6)	0.013	0.330
HDL-c (mmol/L)	1.3 (0.3)	1.3 (0.2)	−0.1 (0.1)	<0.001	1.3 (0.2)	1.3 (0.3)	0.1 (0.1)	<0.001	0.616
LDL-c (mmol/L)	2.9 (0.5)	2.9 (0.5)	0.0 (0.1)	0.986	3.0 (0.7)	3.0 (0.8)	0.0 (0.7)	0.935	0.870

Abbreviations: BMI: body mass index; FPG: fasting plasma glucose; PG: 2-h postprandial plasma glucose; HbA1c: glycosylated hemoglobin; TG: triglycerides; TC: total cholesterol; HDL-c: high-density lipoprotein cholesterol; LDL-c: low-density lipoprotein cholesterol. ^†^ *p-*values within the study group change, based on paired-sample *t*-tests or related samples Wilkoxon Ranks test. ^‡^ *p-*values between groups at the end of study period based on two samples *t*-test or two-sample Mann–Whitney *U*-test.
